# Agricultural Biotechnological Potential of *Bacillus velezensis* C3-3 and *Cytobacillus* sp. T106 from Resource Islands of a Semi-arid Zone of La Guajira-Colombia

**DOI:** 10.1007/s00284-024-03804-8

**Published:** 2024-09-03

**Authors:** Jeimy Daniela Suárez-Bautista, Hillary Sharid Manotas-Viloría, Leslie Leal-Mejía, Johanna Boyacá-Vásquez, Yineth Pineros-Castro, Lucia Constanza Corrales, Laura Cuervo-Soto, Javier Vanegas

**Affiliations:** 1 Colegio Mayor de Cundinamarca University, Bacteriology Program, Cl. 28, #5B-02 Bogotá, Colombia; 2 Department of Biological Sciences, and Sustainable Processes and Products Area Faculty of Natural Sciences and Engineering, Jorge Tadeo Lozano University, Cra 4 # 22-61, Bogotá, Colombia; 3 Department of Biology, Faculty of Sciences, Universidad Antonio Nariño, Cra. 3 Este # 47a-15, Bogotá, Colombia

## Abstract

Resource islands are vegetative formations in arid and semi-arid ecosystems that harbor microorganisms facing extreme conditions. However, there is a limitation in the knowledge of the agricultural biotechnological potential of microorganisms present in these islands. This study aimed to determine the capacity of *Bacillus velezensis* C3-3 and *Cytobacillus* sp. T106 isolates from resource islands to promote plant growth and control the phytopathogen *Rhizoctonia solani*. The bacteria were sequenced, and both grew at 50 °C, resisted 5% NaCl, withstood UV exposure, and grew in extreme pH conditions. Sixty-six genes in C3-3 and 71 in T106 were identified associated with plant growth promotion, and C3-3 was shown to promote leaf growth in lettuce plants. This promotional effect was associated with the production of indole-3-acetic acid (IAA), phosphorus solubilization, and the presence of genes related to the assimilation of rhizosphere exudates. Both strains inhibited *R. solani* through the production of volatile compounds and antagonism. Forty-five and 40 of these genes in C3-3 and T106, respectively, were associated with the production of proteases, lipases, siderophores, antimicrobial compounds, degradation enzymes, and secretion systems. Notably, *Cytobacillus* sp. has not been previously reported as a biocontrol agent. This work contributes to the evidence of the biotechnological potential of semi-arid region bacteria, offering prospects for improving agricultural production in areas with limiting conditions.

**Supplementary Information:**

The online version contains supplementary material available at 10.1007/s00284-024-03804-8.

## Introduction

Arid regions are extremely important ecosystems, housing approximately 30% of the world’s carbon, distributed within the aboveground and belowground biomass, as well as the upper layer of the soil [1]. These regions, which cover more than 41% of the Earth’s land surface and are home to about 2.5 billion people, play a central role in global sustainability [2]. These desert areas also assume a critical role in the present carbon storage, climate change response, and potential to contribute to a carbon–neutral future [1]. However, arid zones grapple with significant desertification processes triggered by climate change and inappropriate land use practices, such as overgrazing, soil degradation, vegetation removal, and deforestation [3]. This desertification process directly affects agricultural growth, poverty reduction and sustainable resource management [4].

In arid and semiarid ecosystems exist islands of resources or fertility, where nurse trees prevail, which promote the establishment of local flora by providing shade and improving the retention of moisture, organic matter, and nutrient content. This catalyzes an increase in microbial activity in the soil [5]. Microorganisms inhabiting these extreme zones have evolved adaptation mechanisms that enable them to withstand harsh conditions, such as low humidity, prolonged exposure to ultraviolet radiation, abrupt temperature fluctuations, and limited nutrient availability [6]. The survival of these bacteria relies on endospore production [7] and the expression of enzymes responding to thermal and oxidative stress [8]. This microbial activity enriches soil quality, notably enhancing carbon retention and nutrient cycling [9]. Moreover, these microorganisms contribute to soil formation and stability through the secretion of exopolysaccharides, bioenzymes, and organic acids [10], while simultaneously establishing beneficial symbiotic relationships with plants.

Plant growth-promoting bacteria (PGPB) are beneficial microorganisms that colonize plant roots and enhance plant growth through various mechanisms. These bacteria have shown remarkable potential to increase agricultural productivity and suppress plant pathogens, making them a promising tool for sustainable agriculture. In arid regions, the presence of PGPB such as *Bacillus subtilis*, *Pseudomonas fluorescens*, *Azospirillum brasilense*, *Acinetobacter* sp., and *Stenotrophomonas maltophilia* has emerged as a critical factor for improving soil quality and plant development under extreme conditions [11]. These microorganisms increase the tolerance of plants to environmental stress and have even proven essential in ecological restoration and the mitigation of desertification in desert environments [12]. For instance, the inoculation of nurse trees with *A. brasilense* and *B. pumilus* in the Sonoran Desert achieved successful revitalization of degraded soil after 11 years of intervention [13].Furthermore, PGPB such as *Acinetobacter*, *Pseudomonas*, *Rhizobium*, *Azospirillum*, *Bacillus*, and *Klebsiella* show significant potential for increasing crop productivity in dry agricultural systems, including wheat, rice, maize, tomato, soybean, and potato [14]. Species of the genus *Bacillus*, such as *B. subtilis, B. licheniformis, B. amyloliquefaciens, B. pumilus* and *B. megaterium*, are particularly effective in controlling phytopathogens [15]. In addition to enhancing plant growth, PGPB such as *B. subtilis*, *P. fluorescens*, *A. brasilense*, *Rhizobium* sp., and *B. amyloliquefaciens* also contribute to improving soil structure and fertility [16]. Other strains like *Halomonas* sp., *Salinibacter ruber, Marinobacter* sp. and *Halobacterium* sp. control phytopathogens through the production of antimicrobial compounds such as siderophores, fluorescent pigments, hydrogen cyanide, glucanases, and chitinases, as well as by increasing the natural resistance of the host [17].

Several strains of *Bacillus velezensis*, such as SQR9 [18], ES2-4 [19], and Q-426 [20], have demonstrated the ability to promote plant growth and inhibit the notorious fungal phytopathogen *Rhizoctonia solani,* which causes significant yield losses in various crops worldwide. These *B. velezensis* strains can stimulate plant growth through mechanisms such as root colonization and the production of secondary metabolites, including phytohormones and siderophores, which enhance nutrient uptake and modulate plant growth [18–20]. These strains can inhibit *R. solani* through the production of antimicrobial compounds, including lipopeptides and lytic enzymes, which disrupt fungal cell walls and suppress pathogen growth. Importantly, PGPR can help plants tolerate various abiotic stresses, such as drought, extreme temperatures, salinity, and heavy metals [21], making them particularly valuable in the context of climate change and soil degradation. However, the biotechnological potential of bacteria in semi-arid environments in the intertropical zone of the Americas remains largely unknown. This zone is characterized by unique ecological conditions, such as limited water availability and high temperatures, which may have shaped the evolution of novel microbial adaptations and traits. Exploring the microbial diversity in these unique environments could lead to the discovery of new PGPB strains with enhanced abilities to promote plant growth, suppress pathogens, and protect plants under challenging conditions, which could have important applications in agriculture and ecosystem restoration in semi-arid and arid regions.

In this context, the objective of this study was to evaluate the capacity of two bacterial strains, *B. velezensis* C3-3 and *Cytobacillus* sp. T106, isolated from a semi-arid region of the Colombian Caribbean, to promote plant growth and exert biocontrol activity. By employing a combination of in vitro assays, in vivo greenhouse experiments, and genomic characterization, this research contributes to the exploration of genomic adaptations that allow these bacteria to thrive in adverse environmental conditions, as well as their practical applicability in agriculture and potential use to halt desertification processes. The characterization of a bacterium belonging to a little-known genus, demonstrates the richness of resource islands as a source of valuable genomic resources for future research aimed at optimizing sustainable agricultural practices in arid ecosystems.

## Material and Methods

### Isolation and Characterization of Bacteria

The sampling site was located at the “Fundación Cerrejón para el Progreso de La Guajira” in the La Guajira Peninsula, situated in the northernmost region of South America (4°38′3.942″N, 74°3′28.206″W). To carry out the isolation and characterization of bacteria, we selected the most prevalent nurse trees in the area identified as *Prosopis juliflora* (Tr), *Pithecellobium dulce* (T), and *Haematoxylum brasiletto* (B), along with a control (C3-3) consisting of bare soil. Twelve soil samples were collected under the canopies of the three nurse tree species and the control, at a depth of less than 2 cm in areas with organic matter present. Each sample consisted of subsamples taken between the trunk of the nurse tree and the edge of the canopy. For the control sample, designated C3-3, soil was collected at a distance of 1 to 2 m from the edge of the resource island, ensuring that it represented the bare soil characteristic of the surrounding area. One gram of soil from each sampling site (T, Tr, B, and C) was enriched in Pseudomonas Agar F (PAF) medium (per liter of water: 10 g proteose peptone, 10 g casein hydrolysate, 1.5 g anhydrous MgSO_4_, 1.5 g K_2_HPO_4_, and 10 mL glycerol) [22].

The isolates were purified and stored at −20 °C in 25% glycerol for further analysis. Of the nine isolated bacteria, isolates C3-3 and T106 were selected due to their rapid and abundant growth in PAF medium after 24 h of incubation, as well as their non-pathogenic nature to humans. The strains C3-3 and T106 were assessed for stress tolerance by growing them in PAF medium under various conditions: acidic (pH 4.5) and alkaline (pH 9.0) environments, high temperatures (50 °C and 70 °C), salt stress (5% and 10% NaCl), and UV radiation exposure (15 mW m^−2^ for 10 min at 50 cm from the UV source). Growth was categorized as scarce ( +), moderate (+ +), abundant (+ + +), or no growth (−).

### Genomic Characterization and Functional Annotation

Genomic DNA was extracted using the DNeasy PowerLyzer PowerSoil Kit from QIAGEN. Library preparation was performed using the NEBNext ultra II DNA Library Prep Kit and the genomes were sequenced using the Illumina NovaSeq6000 platform with a paired-end 2 × 150 bp sequencing strategy. The raw reads were quality-checked using FastQC v0.11.9, and the genomes were assembled using unicycler v0.4.8. The assembled genomes were then annotated using the RASTtk pipeline on the BV-BRC platform v3.28.21 [23]. To assess potential contamination, the 16S rRNA gene sequences were extracted from the assembled genomes and compared against the EzBioCloud database [24] using BLASTN from NCBI [25]. For taxonomic classification of *B. velezensis* C3-3 and *Cytobacillus* sp. T106, the 16S rRNA, as well as the DNA gyrase subunit A and transcription termination factor Rho gene sequences, were extracted from the assembled genomes and aligned against NCBI [25]. Additionally, phylogenetic trees were reconstructed using BV-BRC platform v3.28.21 with a set of 100 conserved single-copy genes, ensuring no duplications or deletions were allowed [23]. Average Nucleotide Identity (ANI) [26] compares the nucleotide sequences of genomes to determine species similarity and assign species identities. The Genome-to-Genome Distance Calculator (GGDC) [27] computes distances between genomes to assess their relatedness. Both methods were used to alleviate taxonomic ambiguity in our study.

The assignment of KO proteins was conducted using the Kyoto Encyclopedia of Genes and Genomes (KEGG) database [28], based on the annotation provided by BV-BRC v3.28.21 [23]. To determine the assignment of KOs in each genome, a list of genes associated with direct and indirect growth promotion mechanisms was constructed employing RAST annotation algorithm with default parameters (gene calling using GLIMMER3, protein translation, and functional annotation based on FIGfams and subsystem collections). The list included, 295 genes related to stress response, 197 involved in colonization (flagella, pili, chemotaxis), 74 associated with the secretion system (Types I, II, III, IV, and V), 30 linked to the assimilation of rhizosphere exudates (amino acids and sugars), 70 related to plant growth promotion (nitrogen fixation, phytohormones, and enzymes), and 150 genes associated with phytopathogens biocontrol (bacteriocins, volatile organic compounds (VOCs), insecticidal activity, and competition for resources).

### 2.3. Biochemical Characterization

#### Indole-3-Acetic Acid (IAA) Production

Indole-3-acetic acid (IAA) production by *B. velezensis* C3-3 and *Cytobacillus* sp. T106 was quantified following the colorimetric method described by Glickmann and Dessaux [29]. Salkowski’s reagent was prepared by dissolving 12 g of FeCl3 per liter in 7.9 M H_2_SO_4_. Bacterial strains were cultured in Luria–Bertani (LB) broth supplemented with 3 mM L-tryptophan. Cultures were incubated at 30 °C with shaking at 180 rpm for 72 h. After incubation, cultures were centrifuged at 10,000 × g for 10 min, and 1 mL of the supernatant was mixed with 2 mL of Salkowski’s reagent. Then, the absorbance was measured at 540 nm using a spectrophotometer after 30 min of incubation in darkness at room temperature. Finally, IAA concentrations were calculated by comparison to a standard curve prepared with commercial IAA (Sigma-Aldrich) at concentrations ranging from 0 to 100 µg/mL and sterile LB broth supplemented with L-tryptophan was used as a negative control.

#### Siderophore Production

Siderophore production was screened using the Chrome Azurol S (CAS) agar assay as described by Schwyn and Neilands [30]. The CAS solution was prepared from three components: (1) 10 mL of 1 mM FeCl_3_·6H_2_O in 10 mM HCl, (2) 50 mL of an aqueous solution of CAS (1.21 mg/mL), and (3) 40 mL of an aqueous solution of hexadecyltrimethylammonium bromide (HDTMA) (1.82 mg/mL). The CAS agar was prepared by mixing 100 mL of the CAS solution with 900 mL of Luria–Bertani (LB) agar, adjusted to pH 6.8 with 50 mM PIPES buffer. The media was autoclaved and poured into Petri dishes. Bacterial strains were spot-inoculated (5 μL of overnight culture) onto the CAS agar plates and incubated at 30 °C for 72 h. Siderophore production was indicated by the formation of orange halos around the bacterial colonies using ImageJ software (version 1.53c, National Institutes of Health, USA) [33]. The diameter of the orange halos was measured after the incubation period. *Pseudomonas aeruginosa* PAO1 was used as a positive control.

#### Phosphate Solubilization

The ability to solubilize inorganic phosphates was evaluated using the method described by Sundara-Rao and Sinha [31]. Sundara-Rao and Sinha (SRS) agar was prepared containing (per liter): 10 g glucose, 5 g (NH_4_)_2_SO_4_, 1 g MgSO_4_·7H_2_O, 0.1 g yeast extract, 15 g agar, and 5 g Ca_3_(PO_4_)_2_ as the insoluble phosphate source. The pH was adjusted to 7.2 before autoclaving. Bacterial strains were spot-inoculated (5 μL of overnight culture) onto the SRS agar plates and incubated at 30 °C for 7 days. Phosphate solubilization was indicated by the formation of clear zones around the bacterial colonies. The diameters were measured after 3, 5, and 7 days of incubation using ImageJ software (version 1.53c, National Institutes of Health, USA) [33]. *P. aeruginosa* PAO1 was used as a positive control and an uninoculated SRS agar plates served as negative controls.

#### Protease Activity

Protease activity was evaluated using skim milk agar (SMA) as described by Vijayaraghavan and Vincent [32]. The SMA was prepared by mixing 10% w/v skim milk powder with 2% w/v agar in distilled water. The mixture was autoclaved at 121 °C for 15 min and poured into Petri dishes. Bacterial strains were spot-inoculated (5 μL of overnight culture) onto the SMA plates and incubated at 30 °C for 48 h. Protease activity was indicated by the formation of clear hydrolysis zones around the bacterial colonies, resulting from casein degradation. After incubation, the plates were flooded with 1% v/v HCl solution to enhance the contrast between the clear zones and the opaque medium. The diameters of the colonies and the clear zones were measured using ImageJ software (version 1.53c, National Institutes of Health, USA) [33]. Uninoculated SMA plates served as negative controls.

#### Lipase Production

Lipase production was assessed using M9 minimal medium supplemented with 1% w/v Tween 20 as described by Al Mohaini [34]. The M9 minimal medium was prepared containing (per liter): 6 g Na_2_HPO_4_, 3 g KH_2_PO_4_, 0.5 g NaCl, 1 g NH_4_Cl, 0.24 g MgSO_4_, 0.011 g CaCl_2_, and 15 g agar. After autoclaving, the medium was supplemented with filter-sterilized 1% w/v Tween 20. Bacterial strains were spot-inoculated (5 μL of overnight culture) onto the prepared agar plates and incubated at 30 °C for 72 h. Lipase activity was indicated by the formation of opaque precipitation zones around the bacterial colonies, resulting from the precipitation of calcium salts of the fatty acids released by the hydrolysis of Tween 20.

### Promotion of Lettuce Seedling Growth

Greenhouse trials were conducted to assess the plant growth-promoting effects of the bacterial strains. The experiments were carried out under controlled conditions with temperatures ranging from 20 °C to 25 °C and natural light. For the plant growth promotion assay, the strains grown overnight in PAF medium were adjusted to different optical densities (0.2, 0.5, and 0.9 at 600 nm absorbance) with Hoagland solution [33]. Subsequently, the roots of 30-day-old lettuce seedlings (*Lactuca sativa* var. Batavia) were immersed for one hour in 200 mL of each bacterial inoculum (12 seedlings per treatment). The seedlings were then transplanted into black plastic bags, each containing 1 kg of non-sterile soil with the following physicochemical characteristics: organic matter content of 117,600 kg, micronutrients in g: sulfur 51.17, boron 0.46, iron 247.70, zinc 5.25, and available potassium 2.6 × 10^4^. Plants were irrigated with 100–200 mL of Hoagland solution per plant every week to maintain optimal growth conditions. Uninoculated plants served as controls. After four weeks of plant-microorganism interaction, chlorophyll concentration was determined in SPAD units using a Portable Chlorophyll Meter CM-A (Shandong, China), and stem, root, and leaf area length were measured using ImageJ software [33]. Additionally, dry weight of the stem and root was determined after drying the plant material at 65 °C for 4 days. Statistical analysis of inoculation effects was performed through analysis of variance (ANOVA) using RStudio version 1.4.1103.

### Biocontrol Trials Against *R. solani*

The bacteria were subjected to two distinct assays to evaluate their antagonist potential against *R. solani:* a first assay based on antagonism and a second assay involving the production of VOCs following the methodology proposed by Azeem et al. [35]. As a control, *R. solani* was cultured without the presence of bacteria. After a three-day incubation period at 28 °C. For the second assay, the inhibition of *R. solani* through the emission of VOCs was evaluated by co-cultivating *R. solani* on PDA, while strains C3-3 and T106 were cultivated on nutrient agar. The cultures were confronted for four days at 28 °C, following the protocol established by Giorgio et al. [36]. The fungal growth diameter was measured using ImageJ software [33], and the percentage of fungal growth inhibition rate (IR) was quantified according to the approach described by Giorgio et al. [36] with the following formula: IR% = 100 × [(C-B)/C], where C is the diameter of the control fungal mycelium and B the diameter of the fungal mycelium grown in the presence of the antagonistic bacteria [36]. Statistically analyzed using one-way ANOVA with a significance level of 0.05. All assays were performed in triplicates, referring to independent biological assays conducted separately to validate our findings.

### Data Availability

Raw sequencing reads were deposited in the NCBI platform at the genBank database under BioProject PRJNA1127043 for *Bacillus velezensis* C3-3 and PRJNA1127038 for *Cytobacillus* sp. T106.

## Results

### Bacteria Isolation

Strains C3-3 and T106 were selected based on their abundant growth on PAF medium at 24 h, their non-pathogenic nature, and their ability to grow under various stress conditions. Both isolates exhibited optimal growth across a wide range of pH values, with C3-3 growing at acidic pH (4.5) and T106 growing at alkaline pH (9.0) and showed abundant growth at 50 °C. Also, T106 exhibited additional thermal tolerance by growing at 70 °C and salinity tolerance by growing under 5% NaCl stress, while C3-3 tolerated up to 10% NaCl concentration. Both isolates demonstrated resistance to UV radiation exposure (Table 1). Gram staining revealed that both C3-3 and T106 are Gram-positive rods. On PAF medium, the colonies of C3-3 appeared white, irregular, and branched, whereas those of T106 were smaller, red, and exhibited regular borders.

**Table 1:** Bacterial growth of *B. velezensis* C3-3 and *Cytobacillus* sp. T106 at different temperatures, salinity, UV and pH

Strain	Temp °C		Stress conditions (50 °C)				
	50	70	NaCl 5%	NaCl 10%	UV 10 min	pH 4.5	pH 9.0
C3-3	+	−	+	+ +	+	+	−
T106	+	+	+ + +	−	+	−	+

Bacterial growth: scarce ( +), moderate (+ +), abundant (+ + +) and no growth (−)

#### Genomic Characterization

**Fig. 1:**
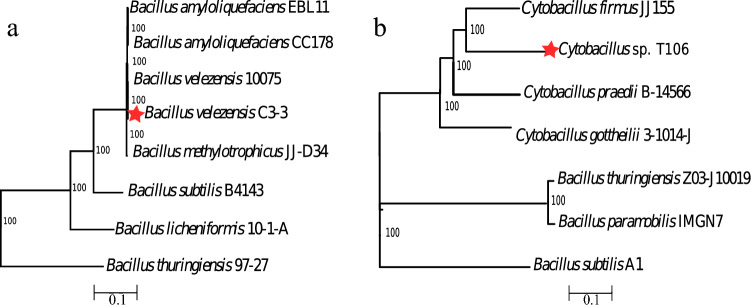
Phylogenetic tree of a. *B. velezensis* C3-3 and b. *Cytobacillus* sp. T106. The percent numbers at the nodes indicate the level 100 genes analyses. The scale bar indicates 0.1 nucleotide substitutions per nucleotide position

Genome assembly revealed that C3-3 and T106 consist of 41 and 44 contigs, respectively. The genome of C3-3 reported 78 tRNA genes, two rRNA genes, and a total of 4051 CDS, including 742 hypothetical proteins, with a size of 3964 Kbp. On the other hand, T106 presented 70 tRNA genes, two rRNA genes, and a total of 5440 CDS, including 2454 hypothetical proteins, with a size of 4955 Kbp. Notably, plasmid information was not detected in these genomes (Fig. 1). To assess the quality and completeness of the assembled genomes, several metrics were evaluated in BV-BRC platform v3.28.21 through assembly and annotation service. For C3-3, the coarse consistency was 99.1%, and the fine consistency was 98%. The CheckM analysis revealed a completeness of 100% and a contamination level of 0.1%. For T106, the coarse consistency was 96.6%, and the fine consistency was 94.3%, with a CheckM completeness of 100%.

**Fig. 2:**
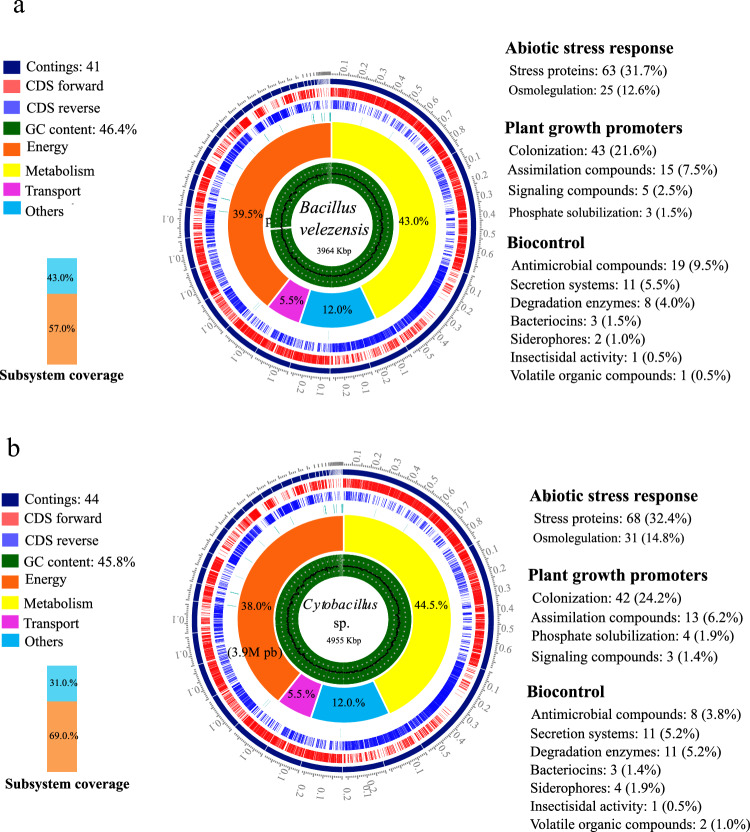
Functional Annotation of the Strains. **a** *B. velezensis* C3-3 and **b** *Cytobacillus* sp. T106

The taxonomic identification of strain T106 using sequences of 16S rRNA, gyrase subunit A (*gyr*A), and transcription termination factor *rho* showed the highest similarity to *Bacillus velezensis*, with sequence identity percentages of 100%, 99.88%, and 99.77%, respectively, and E-values of zero. While *Cytobacillus* sp. T106 reported 97.75% identity with *Cytobacillus* sp. WCC 4585 in the 16S rRNA sequence alignment. However, the alignment of *gyrA* and *rho* found no significant similarity in BLAST within the genus *Cytobacillus*. Phylogenomic analyzes consistently clustered the genome of strain C3-3 with other strains of *B. velezensis* (Fig. 2a), T106 while was located in a robust clade (100% bootstrap support) with species of the genus *Cytobacillus* (Fig. 2b).

ANI analysis revealed that strain T106 exhibited values below 90% when compared to the type strains of *C. firmus* NBRC 15306 (70.83%), NCTC10335 (70.83%), *C. praedii* FJAT-25547 (69.30%), and *C. gottheilii* FJAT-2394 (69.15%). In addition, GGDC analysis showed that strain T106 exhibited values below 70% when compared to the type strains of *C. praedii* ASM143960v1 (19.60%), *C. gottheilii* ASM1858266v1 (21.80%), *C. firmus* ASM3012334v1 (20.70%), and *C. firmus* 50681_C01 (21.80%). In contrast, C3-3 exhibited values above 90% for ANI when compared to the type strains of *B. amyloliquefaciens* ASM1939692v1 (94.90%), *B. velezensis* ASM76955v1 (97.70%), and *B. amyloliquefaciens* DSM 7 = ATCC 23350 ASM19673v1 (95.60%). Additionally, C3-3 showed GGDC values above 70% when compared to the type strains of *B. amyloliquefaciens* ASM1939692v1 (84.90%), *B. velezensis* ASM76955v1 (97.70%) and *B. amyloliquefaciens* DSM 7 = ATCC 23350 ASM19673v1 (95.60%).

#### Functional Annotation

In the C3-3 sample, 199 genes were identified, whereas 210 genes were detected in T106. In relation to common genes associated with stress response, 88 were found in C3-3 and 99 in T106 (Table 2) and genes associated with colonization processes such as flagellum, biofilm, pili, integrases, lipopolysaccharides and chemotaxis, were 39 in C3-3 and 47 in T106 (Table 3). As for the assimilation of rhizospheric exudates of amino acids, sugars, polyamines and citrulline, 15 were found in C3-3 and 13 in T106. Both bacteria have 11 genes associated with type II and V bacterial secretion systems (Vc). Genes associated with plant growth promotion, 6 in C3-3 and 7 in T106 related to phosphorus solubilization and AIA production. In C3-3, we also detected 2 genes responsible for aminobutyric acid aminobutyric acid (GABA) production (Table 3). However, no genes associated with biological nitrogen fixation and 1-aminocyclopropane-1-carboxylate (ACC) deaminase production were detected in either strain.

**Table 2:** Mechanisms involved in the stress response of isolates B. velezensis C3-3 and Cytobacillus sp. T106

Processes	Number of genes in	
	C3-3	T106
Osmoregulation. CT: S-adenosylmethionine decarboxylase, glycine betaine/proline transporter (3), osmoprotectant transporters (3), proline (3), tetraprenyl-beta-curcumin synthase, trehalose (2), other osmoregulators (5), other enzymes (2) and potassium transporter (3)C (7)T. C: glutamate (2) T: spermidine transporter (4)	25	31
Stress proteins. CT: catalase, chaperone (8), dehydrogenase (2), glutathione, heat shock (3), oxidase, peroxiredoxin, esporulation, starvation (2), thioredoxin, transcripcional regulator (4), proteases (11), RNA polymerase (4)C (5)T, other transporters (9)C (10)T, other enzymes (8)C (9)T and others (6)C (7)T. T: glutaredoxin	63	68

Protein/enzyme without parentheses are represented once (1), others, represented with the number in parentheses. C: C3-3, T: T106, CT: C3-3 and T106

**Table 3:** Mechanisms involved in plant growth promotion of the isolates B. velezensis C3-3 and Cytobacillus sp. T106

Processes	Number of genes in	
	C3-3	T106
Amino acids assimilation. CT: arginine descarboxylase, L-asparaginase, L-Glu epimerase, glutaminase and branched-chain aminotransferase. C: D-serine deshydratase	6	5
Sugar assimilation. CT: L-arabinose isomerase (Ara), UDP-glucose 4-epimerase, mannose-6-phosphatase isomerase, xylose isomerase. C: oxalate decarboxylase. T: beta-galactosidase	5	5
Polyamine assimilation. CT: polyamine aminopropyltransferase and spermine N-acetyltransferase	2	2
Assimilation of other compounds: CT: citruline-aspartate ligase. C: betaine aldehyde dehydrogenase	2	1
Signaling enzymes. C: GABA aminotransferase and succinate semialdehyde dehydrogenase	2	0
indole acetic acid. CT: tryptophan alpha-chain synthase, tryptophan synthase beta chain and anthranilate synthase component	3	3
Phophate solubilization (Gluconic acid): CT: gluconate supporter- H + . C: 2-ketogluconate reductase	2	1
Phophatases. CT: alkaline phosphatase synthesis sensor. T: polyphosphate kinase and exopolyphosphatase	1	3
Chemotaxis*. CT: acceptance of methyl, chemotaxis proteins (3), two-component system proteins (4) and purine-binding chemotaxis protein	9	9
Flagella proteins*. CT: biosynthetics (6), M-ring (6), flagellar proteins (5), basal body (5), motor switching (3), RNA polymerase, L-cystine transport, assembly, flagellin and ATPase synthase. T: hook-length control protein	30	31
Pili*. T: Type IV pilus assembly protein (6) and twitching motility protein	0	7
Lipopolisacaridos*. CT: Assembly protein and polyisoprenyl-phosphate glycosyltransferase	2	2
Integrasas*. CT: recombinase (2)	2	2

Protein/enzyme without parentheses are represented once (1), others, represented with the number in parentheses. C: C3-3, T: T106, CT: C3-3 and T106

^*^Represents processes associated with colonization

C3-3 and T106 genomes revealed differences in the abundance of genes related to antimicrobial compounds and degradation enzymes (Table 4). C3-3 possessed a higher number of genes (19) associated with antimicrobial compounds, such as macrolactin, bacillibactin, dificidin, and superoxide dismutase, compared to T106 (8 genes). In contrast, T106 exhibited a greater abundance of genes (11) encoding degradation enzymes, including lipases and metalloproteases, than C3-3 (8 genes). Both isolates harbored an equal number of genes (3) for bacteriocins, specifically amilocyclin and plantazolicin. Notably, C3-3 contained 2 genes encoding the siderophore acromobactin, while T106 possessed 4 genes for the siderophore enterobactin. Additionally, each isolate had a single gene associated with insecticide activity and the production of VOCs (2,3-butanediol for C3-3 and isoamyl alcohol for T106). Both isolates shared a similar genetic composition related to secretion systems, with 9 genes for the type II secretion system and 2 genes for the twin-arginine translocation (Tat) pathway type Vc (Table 4), suggesting their potential role in the secretion of biocontrol-related compounds.

**Table 4:** Pathogenicity mechanisms associated with biocontrol of the isolates B. velezensis C3-3 and Cytobacillus sp. T106

Processes	Number of genes in	
	C3-3	T106
Antimicrobial compounds: CT: macrolactin (2), Bacilano (11)C (2)T, dificidin (4)C (3)T and superoxide dismutase (2)C (1)T	19	8
Degradation enzymes: CT: hemolysins (2), esterase, protease (5)C (6)T. T: lipase, metalloprotease	8	11
Bacteriocins: CT: amilocycin and plantazolicin (2)	3	3
Siderophores: CT: acromobactin (2)T: enterobactin (2)	2	4
Insecticide activity: CT: insecticide toxin	1	1
VOCs: CT: 2,3-butanediol T: isoamyl alcohol	1	2
SS (Tipo II). CT: sec-SRP (9)	9	9
SS (Tipo Vc): CT: protein targeting to the twin translocation arginine pathway (Tat) type Vc (2)	2	2

Protein/enzyme without parentheses are represented once (1), others, represented with the number in parentheses. C: C3-3, T: T106, CT: C3-3 and T106

### Biochemical Characterization

**Fig. 3:**
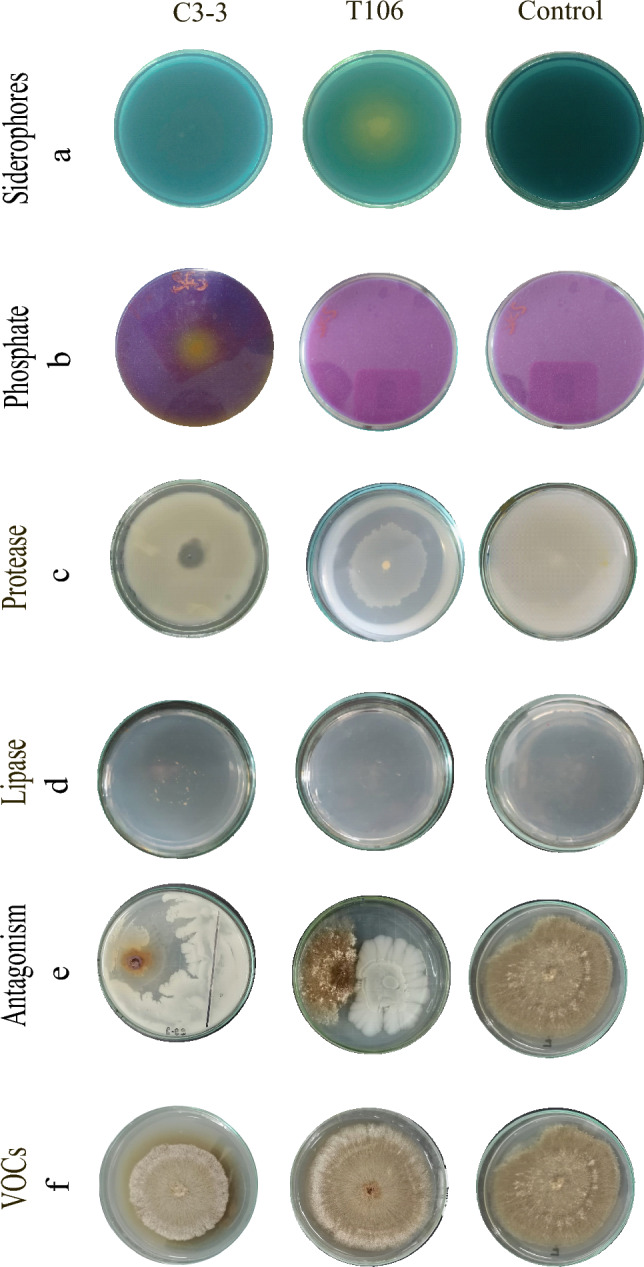
Characterization of strains for growth promotion and biocontrol of *R. solani*. **a** *B. velezensis* C3-3, **b** *Cytobacillus* sp. T106, and **c** Control. Siderophore production was assessed on blue CAS agar, where the formation of a yellow-orange halo around the colonies indicates siderophore production. Phosphate solubilization was evaluated on SRS agar, with the formation of a yellow halo around the colonies indicating phosphate solubilization. Lytic enzyme production, including protease and lipase, was also assessed. The formation of a hydrolysis zone indicates protease production, while precipitates in the agar indicate lipase production

*B. velezensis* C3-3 and *Cytobacillus* sp. T106 were capable of producing IAA, with C3-3 producing 3.78 µg ml^−1^ and T106 producing 6.99 µg ml^−1^. In the protease assay, C3-3 displayed a halo of 2.07 cm, while T106 showed a halo of 1.54 cm. Additionally, C3-3 demonstrated phosphorus solubilization ability and lipase activity, whereas T106 was observed to produce siderophores (Fig. 3, Table 5).

**Table 5:** Biocontrol activity of the strains *B*. *velezensis C3-3* and *Cytobacillus* sp. T106 against *R. solani* and hidrolitic enzymes production

Strains	Antifungal activity (%)		Hidrolitic enzymes	
	Antagonism	VOCs	Proteases (cm)	Lipases
C3-3	34.6	15.2	2.1	+
T106	94.3	34.6	1.5	−

The fungal growth inhibition was quantified using the percentage inhibition. Enzimatic activity: − (no activity), + (activity)

### Promotion of Lettuce Seedling Growth by *B. Velezensis* C3-3

**Fig. 4:**
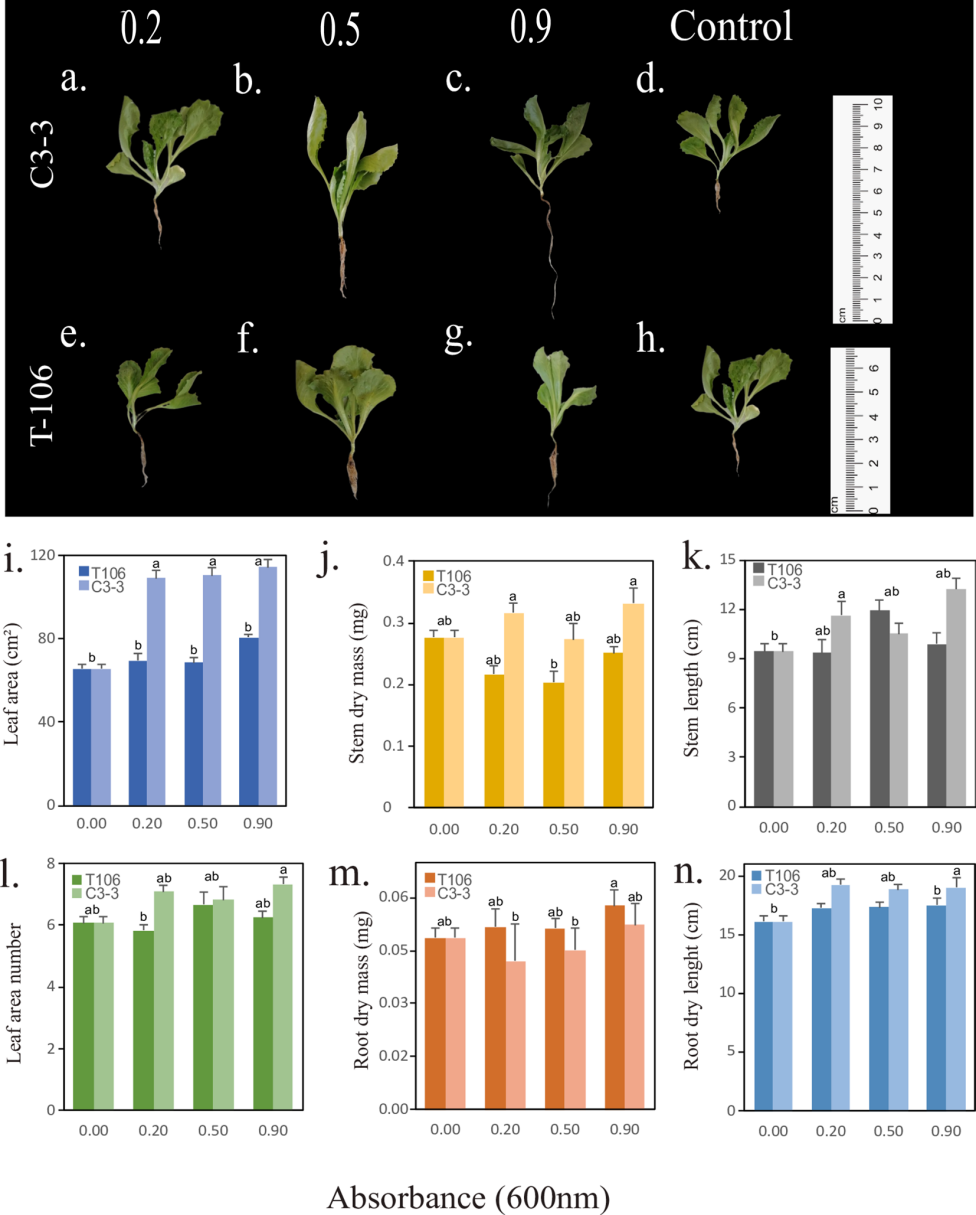
Effect of *B. velezensis* C3-3 and *Cytobacillus* sp. T106 on the growth of Batavia variety lettuce plants (*Lactuca sativa*). Plants were inoculated with bacterial suspensions at optical densities (OD) of 0.2, 0.5, and 0.9, measured at 600 nm. Bars represent the mean values, with error bars indicating the standard error of the mean. Different letters above the bars denote significant differences among treatments, as determined by Tukey's multiple comparison test (p ≤ 0.05)

*B. velezensis* C3-3 at an optical density (OD) of 0.9 showed significant differences in root length compared to the control. At an OD of 0.2, C3-3 significantly promoted lettuce stem growth. The leaf area of lettuce seedlings treated with C3-3 exhibited significant differences at all three absorbance levels tested (0.2, 0.5, and 0.9) compared to the control. In contrast, *Cytobacillus* sp. T106 did not significantly promote plant growth under the tested conditions (Fig. 4).

### Biocontrol Trials Against *R. solani*

Both bacteria inhibited the growth of the phytopathogen *R. solani* by VOCs production (Table 5). The percentage of inhibition (PI) of *R. solani* growth by strain C3-3 was 15.2%, while *Cytobacillus* sp. T106 achieved a PI of 34.6% on the seventh day of confrontation. In the antagonistic activity test, C3-3 exhibited a PI of 34.6%, whereas T106 demonstrated a higher PI of 94.3% against *R. solani* (Table 5).

## Discussion

Resource islands in arid and semi-arid regions represent a rich source of microorganisms with untapped biotechnological potential, particularly for agricultural applications. In this study, we isolated and characterized two bacterial strains, demonstrating their potential to stimulate plant growth in Lettuce (*Lactuca sativa)* and control the phytopathogen *R. solani*. The unique adaptations of these strains to resource island environments may confer advantages over conventional microbial inoculants, especially in arid conditions. These strains have likely evolved to adapt to the extreme conditions prevalent in arid and semi-arid environments, including high temperatures, intense solar radiation, water stress, and nutrient scarcity, potentially conferring unique metabolic capabilities.

### Genomic Characterization

Genome assembly and analysis revealed distinct characteristics for both strains. C3-3 possessed a 3,964 Kbp genome with 4,051 coding sequences (CDS), while T106 had a larger 4,955 Kbp genome with 5,440 CDS. Both genomes lacked plasmids and showed high completeness scores (100%) with low contamination levels, ensuring reliable data for functional predictions. Phylogenetic analyses identified strain C3-3 as *Bacillus velezensis*, a member of the *B. amyloliquefaciens* species complex [37]. This classification was supported by high Average in ANI and GGDC values when compared to type strains, as well as robust clustering in phylogenomic analyses. *B. velezensis* is known for its plant growth-promoting traits and antagonistic activity against various phytopathogens, consistent with our observations.

Strain T106, initial 16S rRNA analysis suggested similarity to *B. infantis*, phylogenomic analyses consistently placed T106 within the *Cytobacillus* clade. However, ANI and GGDC values were well below the species delineation thresholds when compared to known *Cytobacillus* species. These findings suggest that T106 may represent a novel species within the genus *Cytobacillus* or belong to a poorly represented species in existing databases. The genus *Cytobacillus* was recently reclassified from *Bacillus* [38], highlighting the ongoing taxonomic complexity within the *Bacillaceae* family.

### Environmental Stress Resistance

Bacteria inhabiting extreme environments have developed various mechanisms to withstand UV radiation, high-salinity, and elevated temperatures [11]. Genomic analysis revealed 88 and 99 stress-related genes in isolates C3-3 and T106, respectively (Table 2), indicating their potential for stress tolerance. Both isolates demonstrated significant growth at 50 °C (Table 1), likely facilitated by heat-shock proteins (HSPs) encoded by genes such as *hrcA*, *ctsR*, and *HSP20*, as well as molecular chaperones like *dnaK* and *dnaJ* (Table S1). HSPs play crucial roles in bacteria by preventing protein denaturation, assisting in protein folding and assembly, facilitating membrane transport, regulating signaling pathways, inhibiting apoptosis, conferring thermal stability, and ensuring cellular viability under adverse conditions [39, 40]. The moderate growth of T106 at 70 °C (Table 1) can be attributed to genes associated with the synthesis of osmoprotectants and polyamines (Table 2), which activate protective mechanisms against thermal stress [41]. Additionally, the presence of genes encoding glutamate and potassium transporters (*nhaP2*, *kdpB*, *kdpC*, and *kdpA*) suggests the ability to maintain K + homeostasis under hypertonic conditions. This mechanism enhances cellular resistance to elevated temperatures and regulates the accumulation of excess intracellular sodium ions in both bacteria and plants inhabiting saline environments [42].

Both isolates, C3-3 and T106, showed growth in culture media containing 5% NaCl. However, only T106 exhibited growth at 10% NaCl (Table 1). This difference correlates with the number of osmoregulation-associated genes present in each isolate: C3-3 has 25, while T106 has 31 (Table 2). These genes are responsible for the synthesis of osmotic regulators such as proline, trehalose, glycine betaine, and glutamate, which collectively contribute to the isolates’ ability to grow in high-salinity environments. In response to salt stress, the synthesis of osmoprotectants like proline, trehalose, and glycine betaine assists bacteria in maintaining cellular homeostasis by adjusting osmolarity and serving as antioxidants [43]. Additionally, glutamate and γ-aminobutyric acid (GABA) antiporters play protective roles, helping bacteria cope with osmotic, oxidative, and thermal stress [44]. Potassium transporters are crucial in regulating the accumulation of excess sodium ions within bacterial cells in saline environments [45].

The isolates exhibited growth following a 10-min exposure to UV radiation (Table 1), suggesting the presence of UV tolerance mechanisms. In bacteria, particularly within the *Bacillus* genus, these mechanisms typically include the production of protective pigments, DNA repair systems, error-correction proteins, and endospore formation [46]. Genomic analysis revealed that both isolates harbor genes encoding the sporulation sigma factor (*sigH*) and Phase II Q sporulation protein (*spoIIQ*) (Table S1), which facilitate intracellular sporulation [7]. This spore-forming ability likely contributes to their UV tolerance and overall stress resistance.

Additionally, isolates also demonstrated growth under extreme pH conditions (Table 2), indicative of acid and alkali tolerance. pH resilience is typically associated with mechanisms including proton pumps, ammonia production, proton-consuming decarboxylation reactions, modifications in membrane lipid composition, and biofilm formation [46]. The presence of genes *lapA* and *gtrB* in both isolates (Table S2) suggests their capacity for lipopolysaccharide biosynthesis, which may serve as an additional protective mechanism against abiotic stress [47]. Furthermore, both C3-3 and T106 possess genes associated with oxidative stress response, including those encoding superoxide dismutase (Table S3), catalase, and oxidase (Table 2). These enzymes are crucial for protecting bacteria from reactive oxygen species generated under various abiotic stress conditions [8]. The presence of these genes indicates potential to withstand oxidative stress, which is often associated with other environmental stressors such as UV radiation and extreme temperatures.

### Plant Growth Promotion

In a lettuce seedling assay evaluating the plant growth-promoting potential of the isolated bacteria, differential effects were observed between the two strains. The T106 strain showed no significant contribution to plant growth promotion compared to the non-inoculated control. In contrast, the C3-3 strain significantly enhanced root growth, leaf number, and leaf area (*p* < 0.05) (Fig. 2). However, neither strain significantly affected chlorophyll content. The observed enhancement in lettuce growth by the C3-3 strain could be attributed to its higher production of IAA, a well-established plant growth regulator that stimulates root elongation and overall plant development [48]. C3-3 produced 6.99 µg ml^−1^ of IAA, in contrast to T106’s 3.78 µg ml^−1^. This difference in IAA production may explain the contrasting effects of the two strains on lettuce seedlings growth. The plant growth response to bacterial IAA is dose-dependent and can vary among different plant species [49]. While moderate levels of IAA can stimulate growth, excessive concentrations may inhibit plant development. The optimal IAA concentration for growth promotion can differ between species and even between different developmental stages of the same plant [50].

The absence of growth stimulation by T106 in lettuce plants does not necessarily preclude its potential beneficial effects on other plant species. Both C3-3 and T106 possess genes responsible for the synthesis of IAA from tryptophan (*trpA*, *trpB*, and *trpE*) (Table 3) [48], suggesting a shared capacity for auxin production. However, the differential effects observed in lettuce growth promotion indicate that additional factors may influence their plant growth-promoting potential. C3-3 demonstrates a broader range of plant growth-promoting traits compared to T106. Notably, C3-3 presented two genes involved in the synthesis of γ-aminobutyric acid (GABA) (*gabT* and *gabD*) (Table S3) [50]. GABA production has been associated with enhanced plant growth. For instance, *B. velezensis* FX-6, which produces GABA, has been shown to increase biomass in tomato plants by improving essential nutrient absorption, influencing hormone levels that promote cell division, elongation, and differentiation, and enhancing energy production and nutrient utilization [51].

Furthermore, C3-3 exhibited phosphate solubilization activity, a trait potentially attributed to the presence of the gluconate gene *gntP*, an H + symporter involved in the synthesis of organic acids like gluconic acid [52]. The identification of genes *ppk* and *phoR* in C3-3 (Table S2) suggests its capacity to enhance the accumulation of available phosphorus for plants [53]. These genes have been associated with improved phosphorus uptake and plant growth promotion in other *Bacillus* species. For example, *B. megaterium* strain P68, isolated from the potato rhizosphere, significantly increased potato production and phosphorus accumulation through the positive regulation of phosphate transport and related pathways [54].

Colonization plays a pivotal role in the establishment of bacteria within the rhizosphere, facilitating nutrient utilization and ensuring survival in nutrient-poor environments. Genomic analysis revealed that C3-3 and T106 possess 43 and 51 colonization-associated genes, respectively (Table 3), indicating their potential for effective rhizosphere colonization. These genes participate in essential processes such as chemotaxis, motility, adherence, and assimilation of rhizospheric exudates. Both isolates exhibited genes involved in chemotaxis, including *cheA* and *cheY* [55], as well as genes related to flagellar motility such as *flg* and *fli* genes [56]. Additionally, the identification of genes such as *wecB*, *wecA*, *cysE*, *hfq*, *lepB*, and *luxS* provided evidence of biofilm production, with *luxS* specifically associated with the formation of biofilms, motility, and root colonization in *B. velezensis* SQR9 [57]. Regarding T106, genes involved in the biosynthesis and functioning of the pili (*pilA, pilB, pilC, pilM, pilN, pilO*, and *pilT*) [57] were identified (Table S2).

In relation to the assimilation of rhizospheric exudates, 15 genes were found in C3-3, and 13 in T106 (Table 3). This mechanism is linked to the presence of amino acid assimilation genes such as *speA, ansB, ycjG, glsA1*, and *ilvE*. The *speA* gene encodes arginine decarboxylase, a component involved in arginine metabolism that leads to the formation of putrescine, an antioxidant in plants [58]. Additionally, the *ilvE* gene encodes enzymes responsible for the biosynthesis of isoleucine and valine [58]. The *argG* and *speE* genes were also identified, playing a role in the assimilation of citrulline and putrescine (Table S2). These genes are part of the polyamine pathway, which responds to abiotic stress in plants and promotes their growth [58]. The slight difference in gene numbers between C3-3 and T106 suggests potentially distinct strategies for rhizosphere colonization and exudate utilization. Colonization mechanisms are essential for the establishment of bacteria in the rhizosphere, influencing the expression of action mechanisms and generating a positive response to bacterial inoculation.

### Biocontrol

Both C3-3 and T106 isolates demonstrated significant biocontrol potential against *R. solani* through antagonism and VOCs production (Table 4). Genomic analysis revealed 45 and 40 biocontrol-related genes in C3-3 and T106, respectively (Table 5), indicating their robust antimicrobial capabilities. The antagonistic activity of both strains likely involves the secretion of extracellular enzymes (Fig. 3). Proteolytic activity is correlated with genes exhibiting antifungal properties, such as *htpX* [61], *yegQ* [62], and *clp* [63] (Table S3), which were identified in both isolates. The inhibition of fungal pathogens is also potentially associated with siderophore production [64]. While specific siderophore-related genes were not mentioned, the biocontrol efficacy of siderophores like enterobactin in *Pseudomonas aeruginosa* and macrolactin in *Pseudomonas chlororaphis* CP07 against fungi has been well-documented [65].

Genomic analysis showed genes related with the production of other antimicrobial compounds in both strains, including those for bacillane, difficidin, amylomycin, and plantazolicin (Table S3). In *B. velezensis*, these compounds, along with macrolactin, bacilysin, and bacillibactin, have been associated with the inhibition of plant pathogens such as *Agrobacterium* [66] and *Ralstonia solanacearum* [67]. In addition, both strains possess the *budB* gene (Table S3), associated with 2,3-butanediol synthesis, which contributes to antifungal activity and plant growth promotion [60]. Additionally, T106 harbors the *iah1* gene (Table S3), linked to isoamyl alcohol synthesis, a VOC with hydrolytic activity against fungi [60]. These findings align with the known capacity of *Bacillus velezensis* to produce various antifungal VOCs, including 2-nonanone, 2-undecanone, 2-heptanone, and 1-butanol [59].

Other antimicrobial compounds were identified in the isolates as the presence of genes associated with plantazolicin synthesis suggests potential fungicidal activity against *R. solani*, as observed in *Bacillus amyloliquefaciens* [68, 69]. In the secretion system, both bacteria possess nine genes associated with the Sec-SRP secretion systems and two genes in the twin-arginine protein translocation (Tat) pathway (Table S3). Sec-SRP systems enhance biocontrol activity by facilitating the translocation of secretory proteins through the plasma membrane. The Tat pathway is responsible for translocating folded proteins with cofactors across biological membranes. Both pathways contribute to the delivery of virulence factors to host cells during infection [70], potentially enhancing ability to suppress plant pathogens.

This study presents the first report of biocontrol capability of *Cytobacillus* sp. against phytopathogens like *R. solani*. This finding expands our understanding of potential biocontrol agents and opens new avenues for research and application. The comprehensive biocontrol-related gene sets in both C3-3 and T106 suggest their potential as effective biocontrol agents. However, the slight difference in gene numbers between the two strains (45 in C3-3 vs. 40 in T106) warrants further investigation to determine if this translates to differences in biocontrol efficacy across various plant pathogens and environmental conditions.

It is crucial to acknowledge that the response to bacterial inoculation can vary depending on the specific plant species [69] or the presence of different phytopathogenic agents [70]. Furthermore, to validate the specific contribution of each gene to bacterial activities, gene silencing experiments can be conducted targeting the genes of interest in order to elucidate their precise functions. Additionally, a comprehensive chemical characterization of the VOCs produced by these bacteria is recommended to validate compounds that could play a significant role in biocontrol. The exploration of bacteria from extreme environments emerges as an invaluable source of genetic and metabolic resources with potential applications in agriculture, bioremediation, and biomedicine. This research underscores the significance of comprehending the adaptations of these bacteria to adverse conditions and how we can harness their capabilities to address diverse challenges across various fields.

## Conclusions

Resource islands serve as a significant source of bacteria with biotechnological potential. Both *B. velezensis* C3-3 and *Cytobacillus* sp. T106 exhibit genetic adaptations that enable them to survive under abiotic stress conditions, including high temperatures, salinity, UV radiation exposure, and extreme pH levels. Genomic characterization allowed us to identify genes related to stress survival, specifically those involved in the synthesis of heat-shock proteins, production of compatible solutes, spore formation, and encoding antioxidant enzymes. Furthermore, isolates possess genes associated with plant growth promotion and phytopathogen biocontrol, indicating their potential for agricultural applications. Our experimental results demonstrated that *B. velezensis* C3-3 could enhance lettuce growth, possibly through the synthesis of growth regulators like IAA. In contrast, *Cytobacillus* sp. T106 showed ability in biocontrol against *R. solani*, likely due to the production of VOCs, antimicrobial compounds, and hydrolytic enzymes. These findings underscore that the exploration of bacteria from extreme environments represents a valuable source of genetic and metabolic resources with significant potential applications in agriculture, particularly in areas facing similar environmental challenges.

## Supplementary Information

Below is the link to the electronic supplementary material.

Supplementary file1 (XLSX 68 KB)
